# Qualitative study of barriers and facilitators encountered by individuals with physical diseases in returning and continuing to work

**DOI:** 10.1186/s12913-022-08604-z

**Published:** 2022-10-04

**Authors:** Shunsuke Inoue, Seiichiro Tateishi, Arisa Harada, Yasushi Oginosawa, Haruhiko Abe, Satoru Saeki, Junichi Tsukada, Koji Mori

**Affiliations:** 1 Department of Occupational Health Practice and Management, Institute of Industrial Ecological Sciences, University of Occupational and Environmental Health, Japan, Kitakyushu, Japan; 2 Disaster Occupational Health Center, Institute of Industrial Ecological Sciences, University of Occupational and Environmental Health, Japan, Kitakyushu, Japan; 3 Department of Occupational Medicine School of Medicine, University of Occupational and Environmental Health, Japan, Kitakyushu, Japan; 4 The Second Department of Internal Medicine, School of Medicine, University of Occupational and Environmental Health, Japan, Kitakyushu, Japan; 5 Department of Heart Rhythm Management, School of Medicine, University of Occupational and Environmental Health, Japan, Kitakyushu, Japan; 6 Department of Rehabilitation Medicine, School of Medicine, University of Occupational and Environmental Health, Japan, Kitakyushu, Japan; 7 Hematology, University of Occupational and Environmental Health, Japan, Kitakyushu, Japan

**Keywords:** Return to work, Continue to work, Qualitative study, Stroke, Heart disease, Cancer survivors

## Abstract

**Background:**

The number of employees with physical diseases is increasing, and there is a need for support to help them return and continue to work. To provide effective support, it is important to identify barriers and facilitators for individuals in returning and continuing to work. Previous studies have reported barriers and facilitators for specific diseases. However, few reports have dealt with these issues across various diseases. To identify a range of barriers and facilitators that may apply to different physical diseases, we conducted a qualitative analysis by interviewing patients with diverse characteristics being treated for diseases.

**Methods:**

We conducted semi-structured interviews based on the criteria for qualitative research. We investigated three disease groups to obtain details of barriers and facilitators: impairments that were visible to other people (mainly stroke); impairments invisible to others (mainly heart disease); and impairments that changed over time (mainly cancer). Interview transcripts were analyzed and the results reported using systematic text condensation.

**Results:**

We extracted 769 meaning units from 28 patient interviews. We categorized barriers and facilitators that were generalizable to various diseases into three themes (personal factors, workplace factors, and inter-sectoral collaboration and social resources) and 10 sub-themes (work ability, psychological impacts, health literacy, social status, family background, workplace structure, workplace system, workplace support, inter-sectoral collaboration, and social resources).

**Conclusions:**

This study identified 10 sub-themes that can be applied for workers with physical diseases; those sub-themes may be used as a basis for communicating with those individuals about returning and continuing to work. Our results suggest that various barriers and facilitators for workers with physical diseases should be understood and addressed at medical institutions, workplaces, and support sites.

## Background

In developed countries, advances in medical technology have extended healthy life expectancy, and declining birthrates have resulted in later retirement ages among workers. These changes have led to an increase in the proportion of workers with chronic physical diseases [1–3]. Recent reports indicate that about 24% of the working population in Europe and 30% of that in Japan have health conditions for which they need medication [4, 5]. Given the increase in the disease prevalence among the working population, it is important to establish systems in workplaces to support individuals returning and continuing to work. This necessity is consistent with the sustainable development goals (SDGs) set by the United Nations on September 25, 2015. The United Nations sets a target for SDGs of 8.5 by 2030: that amounts to achieving full and productive employment and decent work for all women and men, including for young people and persons with disabilities, and equal pay for work of equal value [6].

It has been reported that barriers and facilitators for individuals to return to or continue working differ among different diseases. For example, barriers and facilitators related to stroke have been reported for gender and age [7–9], presence or absence of function in hemiplegic hand [7, 8], ability to independently perform activities of daily living [7, 8, 10], cognitive capacity [10], adjustments and flexibility in the workplace [11], and support from supervisors, coworkers, and family [11]. For heart disease, identified barriers and facilitators have been related to gender and age [12, 13], recurrent cardiovascular events (e.g., sudden cardiac death) [12, 14], psychological factors [13, 14], and workload [12, 13]. With cancer, barriers and facilitators have included gender and age [15, 16], physical symptoms (e.g., fatigue) [16, 17], psychological factors [16–18], economic difficulties [17, 19], workload [14, 18], job control [20], support from supervisors, colleagues, and family [17, 20–22], and support from occupational health professionals [23].

The intervention of health professionals (e.g., occupational health professionals and vocational rehabilitation staff) is a system to support individuals with chronic diseases returning and continuing to work [24, 25]. These occupational health professionals provide support for the worker’s safety in the workplace, and vocational rehabilitation staff provide support for their health care or activities of daily living. Health professionals are expected to conduct assessments that focus on the symptoms, functions, resources, and barriers and facilitators for individual workers, rather than deciding how to support these employees based on disease pathology alone. These professionals collaborate with various stakeholders (e.g., employers and general practitioners) involved in decisions about returning and continuing to work; they may also interact with employers about the possibility of implementing accommodations (e.g., job rotation and other job accommodations) [24]. However, previous reports have been limited to specific diseases, such as stroke, heart disease, cancer, musculoskeletal disorders [26], and mental health problems [27].

To identify a range of barriers and facilitators that can apply to various types of physical diseases, we conducted a qualitative analysis by interviewing patients being treated for different conditions. The findings could inform policy decisions about facilitating individuals returning and continuing to work.

## Methods

### Design and setting

We chose a qualitative study design using content analysis. A qualitative design can help identify patients’ perspectives based on their expressions by describing, interpreting, and generating theories about social interactions and individual experiences as they occur in natural, rather than experimental, situations [28]. We undertook this study following the consolidated criteria for reporting qualitative research [29] and the standards for reporting qualitative research [28].

In this study, we focused on three disease groups to collect various barriers and facilitators: (1) impairments that were visible to others (e.g., cerebrovascular disease [stroke] with paralysis); (2) impairments invisible to others (e.g., cerebrovascular disease [stroke] with higher brain dysfunction and heart disease with arrhythmia and heart failure); and (3) impairments that changed over time (e.g., cancers). This research was performed by selected specialists (rehabilitation medicine, cardiologists, and hematologists) at our medical institution who were primarily responsible for these diseases. This allowed collection of information in a detailed, methodologically consistent manner. We excluded mental health problems because they are closely related to work. The research group covered several disciplinary backgrounds, including occupational medicine (SI, AH, MY, ST, KM), rehabilitation medicine (SS), cardiovascular medicine (HA, YO), and hematology (JT). All members of the research team had experience in supporting workers in returning and continuing to work.

### Selection of participants and procedure

The target population was defined as workers with stroke, heart disease, or cancer who visited X hospital from March 2015 to March 2016. The eligibility criteria were as follows: (1) residing in the Kitakyushu medical area; (2) working before treatment; (3) working after acute treatment; and (4) over 30 days after returning to work. The exclusion criteria were as follows: (1) unable to return to work; and (2) return to work for a second or subsequent time.

The attending physicians verbally invited all patients who met the above criteria to cooperate in this study; interviews were conducted with 28 patients who consented to participate. There were no interruptions or withdrawals of consent during the study period. The interviews were conducted between April 2015 and March 2017.

### Data collection

We conducted semi-structured interviews in a consultation room in each department after the participants visited X hospital. The interviews were conducted face-to-face by a physician from each department and one member of the research group. Each interview lasted 30 min to 1 h, depending on the case. The interviews were audio-recorded with the participants’ consent. The interviewers made field notes to describe details of the participants’ nonverbal expressions and interview context. Information on the participants’ clinical backgrounds (gender, age, diagnosis, treatment) was obtained in advance from the attending physician based on the participants’ medical information with their consent. The interviewers were trained rehabilitation physicians for stroke, cardiologists for cardiac disease, and hematologists for cancer cases.

### Data analysis

The interview transcripts were analyzed using qualitative content analysis with systematic text condensation, which is a descriptive, cross-case analysis strategy [30]. The analysts (SI, ST, AR, and KM) read all transcripts to obtain an overall impression. Three main higher-order themes were agreed upon as important for the study question: personal factors, workplace factors, and factors for information sharing by hospitals. All texts were imported into Microsoft Excel, divided into clauses, and then supplemented and simplified as much as possible with peripheral information (e.g., the aforementioned explanations designated by indicative words). This allowed the background of the statements, such as character relationships and job descriptions, to be understood using only the divided clauses (SI and AR). To preserve confidentiality, participants were referred to by the letters “s” (stroke), “h” (heart disease), or “c” (cancer) followed by an ordinal number (1–14). We conducted a detailed search for meaning units. Meaning units are short or longer pieces of text that help answer a research question [30]. The identified meaning units were coded and then sorted into sub-themes, which summarized what the meaning units jointly described, and then grouped into larger themes (SI, ST, AR, and KM). The sub-themes were combined with illustrative quotations (SI, ST, and KM). Finally, a descriptive narrative of the sub-themes based on meaning units was provided to contextualize the analysis. Quotations were translated from Japanese to English by the first author and edited by a professional language-editing service.

### Reflexivity

Our study group comprised occupational health professionals and physicians from various medical departments, all of whom were educated in occupational health, including returning and continuing to work. With respect to workplaces and medical institutions, we have considerable experience of individuals with diseases struggle to return and continue to work and wished to identify the related barriers and facilitators.

To standardize our approach, we developed an interview guide and conducted semi-structured interviews. The physician was either the attending physician or person in charge of each medical department (YO, HA, SS, JT). The other members of the study group were occupational physicians and had never previously met the participants. The presence of the attending physicians could have made it difficult for participants to speak openly, which could have compromised objectivity. To avoid this, a researcher who was not the attending physician participated in the interviews and was encouraged to ask the participants questions.

### Trustworthiness

We considered the trustworthiness of the sub-themes and themes to be assured when each of the three researchers presented an idea and the agreement rate was greater than 70%. If the agreement rate was less than 70%, a supervisor was added, and the trustworthiness was repeatedly checked until the agreement rate among the four researchers exceeded 70%.

### Ethics

We did not interview individuals who were unable to return to work because of the potential for trauma. The purpose, methods, publication, and ethical considerations (e.g., protection of personal information) of the study were explained to all participants in written format, and their willingness to participate was confirmed by signing a consent form. Participants were informed that they could withdraw their consent at any time. All the methods were conducted in accordance with relevant guidelines and regulations. This study was undertaken with the approval of the Research Ethics Review Committee of the University of Occupational and Environmental Health (Approval No. H27-002).

## Results

### Participant characteristics

The participant characteristics appear in Table 1. We obtained consent to participate in this study from 28 patients (five with stroke, nine with heart disease, and 14 with cancer); 22 were male, six were female, and the mean age was 53 years (standard deviation, 10 years). The participants had a range of employment status, including civil servants, full-time work, and part-time work. Five participants used cardiac devices, such as cardiac resynchronization therapy defibrillators and implantable cardioverter defibrillators.

**Table 1:** Participants' characteristics

Participant	Age group	Sex	Diagnosis	Treatment	Occupation	Occupational categories	Employment status
s.1	50	Male	Brain infarction and left hemiplegia	Rehabilitation	Photography studio (M)	Blue collar	Self-employed
s.2	60	Male	Brain infarction, right hemiplegia, and motor aphasia	Rehabilitation	Clerk	White collar	Permanent staff
s.3	60	Male	Brain infarction, left hemiplegia, and higher brain dysfunction	Rehabilitation	Clerk	White collar	Permanent staff
s.4	40	Male	Brain infarction, left hemiplegia, and higher brain dysfunction	Rehabilitation	Construction management (M)	White collar	Self-employed
s.5	30	Male	Cerebral hemorrhage, right hemiplegia, and motor aphasia	Rehabilitation	Transportation and sales	Blue collar	Contract staff
h.1	60	Female	Chronic heart failure	CRT-D	Sales clerk	White collar	Permanent staff
h.2	40	Female	Arrhythmia	ICD	Nurse	Blue collar	Permanent staff
h.3	50	Male	Arrhythmia	ICD	Teacher	White collar	Public employee
h.4	50	Male	Arrhythmia	ICD	Teacher	White collar	Public employee
h.5	60	Male	Acute myocardial infarction	PTCA	Sales (M)	White collar	Permanent staff
h.6	60	Male	Arrhythmia	ICD	Instructor (steel industry) (M)	White collar	Permanent staff
h.7	40	Male	Chronic heart failure	Pharmacotherapy	System engineer	White collar	Permanent staff
h.8	70	Male	Acute myocardial infarction	PTCA	Protective fore	Blue collar	Contract staff
h.9	60	Male	Chronic heart failure	Pharmacotherapy	Welder	Blue collar	Permanent staff
c.1	40	Male	Malignant lymphoma	CT	Production line	Blue collar	Permanent staff
c.2	40	Male	Malignant lymphoma	CT	Construction worker	Blue collar	Public employee
c.3	30	Female	Malignant lymphoma	CT and RT	Caregiver	Blue collar	Permanent staff
c.4	50	Male	Multiple myeloma	CT	Prison officer	Blue collar	Public employee
c.5	40	Female	Malignant lymphoma	CT and RT	Construction worker	Blue collar	Permanent staff
c.6	60	Male	Malignant lymphoma	CT	Janitor and clerk	Blue collar	Contract staff
c.7	50	Male	Malignant lymphoma	CT	Construction supervisor (M)	White collar	Permanent staff
c.8	50	Male	Leukemia	CT and BMT	Clerk	White collar	Permanent staff
c.9	60	Female	Leukemia	CT and BMT	Care manager	White collar	Permanent staff
c.10	40	Male	Leukemia	CT and BMT	Paint applicator (M)	White collar	Permanent staff
c.11	40	Male	Malignant lymphoma	CT	Clerk	White collar	Permanent staff
c.12	40	Male	Leukemia	CT and BMT	Construction worker	Blue collar	Permanent staff
c.13	50	Female	Breast cancer	Surgery, CT and RT	Restaurant staff	Blue collar	Part-time
c.14	50	Male	Esophageal cancer	Surgery and CT	Sales (M)	White collar	Permanent staff

*H* Heart disease, *S* Stroke, *C* Cancer,* CRT-D* Cardiac resynchronization therapy-defibrillator, *ICD* Implantable cardioverter defibrillator, *PTCA* Percutaneous transluminal coronary angioplasty, *CT* Chemotherapy, *RT* Radiotherapy, *BMT* Bone marrow transplantation, *M* Manager

### Barriers and facilitators for patients returning to and continuing to work

A minimum of 11 and maximum of 46 meaning units were extracted from each participant. In total, 769 units were extracted: 117 from stroke participants, 227 from heart disease participants, and 425 from cancer participants. We categorized those units into 10 sub-themes (work ability, psychological impacts, health literacy, social status, family background, workplace structure, workplace system, workplace support, inter-sectoral collaboration, and social resources) and three themes (personal factors, workplace factors, and inter-sectoral collaboration and social resources). The extracted themes, sub-themes, barriers, and facilitators appear in Table 2. The content and supporting citations for each sub-theme are presented below.

**Table 2:** List of themes, identified sub-themes as retrieved on interviews

Themes	Sub-themes	Barriers			Facilitators		
		Strokes	Heart Diseases	Cancers	Strokes	Heart Diseases	Cancers
		(*n* = 5)	(*n* = 9)	(*n* = 14)	(*n* = 5)	(*n* = 9)	(*n* = 14)
Personal factors	Work ability	16	21	79	13	21	5
	Psychological impacts	8	20	20	7	17	8
	Health literacy	0	2	5	6	16	38
	Social status	2	3	2	6	3	16
	Family background	1	3	16	2	11	8
Workplace factors	Workplace structure	5	14	20	5	6	20
	Workplace system	2	4	6	5	13	33
	Workplace support	3	12	15	19	43	80
Intersectoral collaboration and social resources factor	Intersectoral collaboration	1	0	13	0	14	16
	Social resources	5	2	13	11	2	12

#### Personal factors

The sub-themes extracted were work ability, psychological impacts, health literacy, social status, and family background. For work ability, there were three identified barriers: symptoms, treatment side effects, and disability. With symptoms, we observed that the more severe an individual’s symptoms, the more severe the barriers tended to be. The impact of indirect symptoms (such as decreased physical strength and fatigue) and risk of occupational accidents (e.g., in patients with cardiac devices) also emerged. Facilitators included medical rehabilitation and voluntary training to restore disability and impairment, thereby improving work ability.

*The problem I have at work is my left hemiplegia...I can’t change a 5-kg roll in the darkroom, and I can’t do fine work. I can’t hold the camera when I take a picture. I can’t even set up a tripod. I can’t press the shutter with too much force...If I do it too many times, it bothers the customers (photography studio manager, male, 50 years).*

*Owing to hip joint pain caused by cancer, it is hard for me to move around, such as going up and down stairs. Also, the side effects of the chemotherapy make me very tired, so I can’t do any work that involves moving heavy things (construction worker, male, 40 years).*

*I had a stroke and was told by the doctor that I would be bedridden at first. However, I wanted to walk, so I worked very hard in rehabilitation and became able to walk with a cane. As a result, I can move around to workplaces that I couldn’t enter with a wheelchair (photography studio manager, male, 50 years).*

Regarding psychological impacts, barriers included negative emotions and spiritual pain, such as the following: fear of relapse and treatment; stigma of being called physically handicapped; feeling forced to discontinue work because of disease; loss of motivation to work due to changes in appearance; and decreased confidence in one’s own work. Those barriers enhanced a state of reluctance to return and continue to work. Facilitators for a positive attitude to work were being able to overcome negative emotions, handling personal feelings, and gaining spiritual growth by accepting the disease.

*The problem I had when I returned to work was anxiety. I was given a pacemaker, but I was worried that I might have another attack at work (teacher, male, 50 years).*

*I have the same job as any normal person, but because I have a disability certificate, that makes me disabled. I don’t want to think of myself as a disabled person: I feel like a healthy person now I have a heart device (salesclerk, female, 60 years).*

*At work, I think my attitude toward the elderly and staff has changed. When an elderly woman tells me that she has chest pains, I can sympathize with her and say, “It’s painful, isn’t it?” That’s why I’m glad I became ill. I think my sense of values has changed for the better, both in terms of work and life (nurse, female, 40 years).*

For health literacy, barriers included lack of ability to obtain, understand, and apply accurate information about health and medical care. Inability among participants to help themselves owing to lack of knowledge and motivation was a barrier to returning to work. Facilitators included participants’ being able to recognize their own issues, acquire information, and use that information for self-care. Those who were able to recognize and address their own issues were able to return and continue work more smoothly.

*I didn’t look up any information about the disease. I had a headache and felt it would interfere with my work. But I judged it would be like that after anticancer drugs and didn’t take any action (construction worker, male, 40 years).*

*I was aware of my declining physical strength, probably because I’d spent 2 months in the hospital and recuperating at home. Even with reduced work, I became easily tired and had trouble continuing work. So I researched ways to cope on my own, consulted with my doctor, started walking and taking other moderate exercise, and worked hard to build up my physical strength (teacher, male, 50 years).*

Regarding social status, barriers included economic difficulties, employment, professionalism, and other attributes specific to the individual. Participants felt obliged to return to work following inadequate medical treatment, which made it difficult for them to continue working. Facilitators included receiving support payments (such as salary compensation during leave), qualifications and expertise, and stable employment. Those stable factors made participants ready to return to work and facilitated continuing to work.

*As a public employee, I could take leave for a certain period of time, and I could concentrate on dealing with my disease first. I was financially compensated, so I wasn’t worried about returning and continuing to work (construction worker, male, 40 years).*

For family background, barriers included participants’ feeling that they had to play a role in the family and their family demanding that they perform limited work. Participants had returned to work despite the difficulties they felt in continuing to work. Facilitators were family members that were able to accept and consult with the participant about their condition and provision of support for daily life activities, such as commuting*.*

*I was able to renew my driver’s license after my stroke, and my doctor allowed me to drive. But my family was against me driving, so I couldn’t do my sales job, which required a car (clerk, male, 60 years).*

#### Workplace factors

The sub-themes identified were workplace structure, system, and support. Structure relates to circumstances that could not be changed by the participants, such as equipment and location; system signifies elements that are not unchangeable but difficult to modify, such as regulations; support is the most flexible of the three sub-themes and includes support from supervisors and coworkers.

Barriers in the workplace structure were company-dependent structural problems that could not be solved or changed by participants (e.g., workplace location and lack of personnel) and environmental problems (e.g., dusty workplaces and machines causing electromagnetic interference with cardiac devices). Special environments that exacerbated preexisting conditions made it difficult for participants to return to work. Such factors as inconvenient location and understaffing prevented them from continuing to work. Facilitators included having resting facilities, sufficient staff, and an occupational physician. They helped participants continue working.

*My workplace is far from the station and bus stop. If they were closer, I could walk to work, but it’s impossible at my current workplace, and I have trouble commuting (clerk, male, 60 years).*

Regarding workplace system, barriers included inadequate regulations and systems for returning to work and inadequate adjustments and flexibility in the workplace. They constituted barriers because they were not designed to expedite return and continuing to work. Facilitators included existing regulations for reinstatement and having discretionary authority about work: they were such factors as workplace rules or culture that made it easier for participants to work.

*The system for returning to work was well organized, and there was sufficient time for sick leave so I could receive good treatment (clerk, male, 40 years).*

For workplace support, barriers were interpersonal factors related to inappropriate or poor support from supervisors and colleagues. In the early stages of returning to work, the amount of work that participants could do tended to be lower than when they were in good health: lack of coworker support made it difficult for participants to perform their work. Facilitators included being approached in a supportive manner by supervisors and colleagues and being listened to. Accommodation was also included in this sub-theme: it helped them do work despite difficulties in returning and continuing to work.

*When I was just discharged from hospital, my boss took over my work and said I didn’t have to do driving duties. After that, my boss kept asking me how I was doing. When I asked for advice, he listened (janitor and clerk, male, 60 years).*

#### Inter-sectoral collaboration and social resources factors

Regarding inter-sectoral collaboration, barriers included over- or under-sharing medical information necessary for an individual returning and continuing to work between the medical institution and workplace. Over-sharing of information can be misused by an employer. Under-sharing information prevents the workplace from providing appropriate support. Facilitators included support of the attending physician and the medical institution and occupational physician working together to share information. If these collaborations are appropriate, the patient’s situation in the workplace can be accommodated, facilitating return and continuing to work.

*My doctor wrote a letter to my workplace, detailing the symptoms of my fatigue, the treatment schedule, and tasks that needed attention. This helped the people at work understand me (caregiver, female, 30 years).*

For social resources, barriers included difficulties in accessing and using information on leave programs, salary compensation programs, and medical expense caps. Lack of financial and patient support caused difficulties in returning and continuing to work. Facilitators were being able to access and use social resources, such as various support systems and opportunities to talk with peer supporters. This information was often provided by workplaces, medical institutions, and friends. Receiving financial and patient support and being able to focus on medical treatment and share information promoted returning and continuing to work.

*I didn’t get any compensation during my leave. I didn’t know about such compensation. I didn’t know about it until my friend informed me. If I’d gotten the information earlier, I wouldn’t have been in trouble (clerk, male, 60 years).*

## Discussion

In this study, we identified three themes (personal factors, workplace factors, and inter-sectoral collaboration and social resources) and 10 sub-themes (work ability, psychological impacts, health literacy, social status, family background, workplace structure, workplace system, workplace support, inter-sectoral collaboration, and social resources).

### Consideration of themes

#### Personal factors

Our results related to sub-themes identified in the personal factors theme are similar to those of previous reports in terms of work ability, psychological impacts, social status, and family background. Other studies recognized work ability as a factor that affected individuals returning and continuing to work following various diseases, including stroke, heart disease, and cancer [31–34]. Notably, the presence of comorbid conditions and chemotherapy have been recognized as barriers that affect an individual returning and continuing to work and reduced their work ability [31, 32]. We observed that work ability decreased through such issues as paralysis and chemotherapy side effects (barriers), whereas effective rehabilitation enabled participants to recover to the extent that they could perform their jobs (facilitator).

Previous studies have reported the psychological impacts that affect individuals returning and continuing to work in the context of specific diseases [13, 14, 16–18, 35]. Identified barriers were negative emotions, such as lack of confidence, anxiety, and depression. In this research, we did not focus on the presence or absence of depression, but we identified negative emotions, such as lack of confidence and spiritual pain (e.g., “I don’t want to think of myself as disabled”), as barriers. Our participants recognized that they were able to overcome these negative feelings and gained spiritual growth by accepting their disease, which were considered facilitators.

Social status is reportedly a factor that affects returning and continuing to work with specific diseases [7, 13, 17, 19]. Identified barriers were old age, low education, and economic difficulties [7, 17, 19]; a major facilitator was employment stability [13]. In this study, we did not extract statements about age and education, but we identified barriers related to economic difficulties and employment; we found employment stability and having expertise to be facilitators.

Other research has found family background to be a factor affecting returning and continuing to work with certain diseases [11, 21, 22, 36, 37]; an overprotective family [22] and having dependents [37] have been identified as barriers. Similarly, we observed lack of family support, overprotection, and having dependents to be barriers to returning and continuing to work.

A major difference between the findings of previous studies and ours concerned health literacy. This factor was considered important in treatment [38]; however, there are no reports of health literacy as a factor affecting returning or continuing to work among individuals with physical diseases. Health literacy has been defined as “the degree to which individuals have the capacity to obtain, process, and understand basic health information and services needed to make appropriate health decisions” [39]. One study of heart disease suggested that low health literacy may be a barrier to effective disease self-management [40]. In the present investigation, such factors as not obtaining disease information and not processing symptoms even if they were troubling were identified as barriers; recognizing one’s own issues, obtaining information, and using that information for self-care appeared as facilitators. One reason for this difference among studies may be that most of the attending physicians in our study were experienced occupational physicians. As a result, 60 items were reported as factors promoting health literacy. However, it is important to note that there were seven items that were identified as barriers—even though health literacy is often taught in regular practice.

#### Workplace factors

In this study, similarities with previous research were evident in the workplace system and support sub-themes. Workplace systems were considered a factor affecting returning and continuing to work in studies focused on specific diseases, such as regulations for returning to work, adjustments and flexibility at the workplace, and job control [11, 12, 20, 41]. We identified similar factors in all three disease groups. Many studies have reported that support from supervisors and coworkers (workplace support) may be facilitators for an individual with a physical disease returning and continuing to work; lack of this support is a barrier [13, 17, 18, 20, 23, 36, 42, 43]. Our study confirms workplace support as a factor for all disease groups.

In terms of workplace structure, one study of chronic diseases identified the absence of an occupational physician as a barrier [44]. In an investigation of cancer, company size emerged as a barrier [45]. For all disease groups, we identified structural factors, such as workplace size (small), location (far from train or bus access), and shortage of workers as barriers. Work environment factors (e.g., hot workplaces and equipment affecting cardiac devices) were a barrier for cardiac disease, and dusty workplaces were a barrier for cancer patients.

#### Inter-sectoral collaboration and social resources factors

Similarities between our study and other research emerged in terms of social resources. One investigation of chronic diseases recognized the difficulty of using social security; lack of information about this resource was a barrier to an individual continuing to work [41]. In our study, facilitators related to this issue were being able to obtain and use information on social resources (such as compensation for absence when participants returned to work); barriers were being unable to obtain or use such information. There is a discrepancy between reports suggesting that leave and salary compensation systems promoted return to work [46] and those that found no such effect [47]. In the United States, failure to return to work results in loss of health insurance; thus, maintaining health insurance was identified as a facilitator for returning to work [46]. In contrast, with the Dutch disability policy, the return-to-work rate of breast cancer patients decreased when the compensation for absence from work was extended from 1 to 2 years [47].

One study that considered the perspective of occupational rehabilitation clinicians found coordination of activities and dialogue with key stakeholders to be an important inter-sectoral collaboration factor for returning to work [24]. From the patients’ perspective, we found that coordination between medical institutions and workplaces to be a facilitator; the failure of such coordination was a barrier. In some situations, providing information about health status could be misused by employers. To avoid this, collaboration should always be conducted with the consent of the individual. Workplaces should limit who handles such information and declare that it will not be used for any purpose other than to support returning and continuing to work. Medical institutions should target professionals with confidentiality obligations, such as occupational health professionals, as collaboration partners.

### Strengths and limitations

A limitation of this study was that we conducted it at only one medical institution; although we selected three physical disease groups to allow evaluation independent of disease, selection bias may have occurred. We selected subjects by focusing on the following impairments irrespective of disease: (1) impairments that were visible to others; (2) impairments invisible to others; and (3) impairments that changed over time. Therefore, our results could be adapted to physical diseases that we did not consider as long as they meet our criteria. However, there are limitations to generalization. It is necessary to note that our results do not relate to all diseases. In addition, our study focused on physical diseases; psychiatric diseases demand separate examination.

We were unable to assess cases in which employees were unable to return to work because of ethical considerations. Some characteristics of the Japanese labor market need to be considered when adapting our findings to situations overseas. The characteristics of Japan’s labor market are as follows. (1) Among organisation for economic co-operation and development (OECD) countries, Japan has the largest number of workers aged 55 years and above (in 2020, 22% on average for the OECD versus 31% for Japan) [48]. With Japan’s declining working population, it is necessary to support people who are willing to work; thus, there is a need to implement effective systems in the workplace. (2) Japan has a universal health insurance system, which features a monthly co-payment threshold for expensive medical treatment. With that insurance system, certain benefits are provided in the case of unemployment.

## Conclusions

**Fig. 1:**
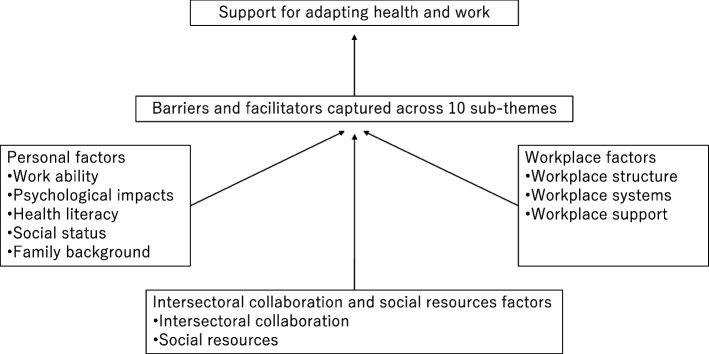
Practical implications of the 10 sub-themes

This study identified 10 sub-themes that can be applied for workers with physical diseases; we believe those sub-themes can provide a basis for communication in promoting individuals returning and continuing to work. For individuals with physical diseases who are able to return to and continue working, it is necessary to reduce the discrepancy between their health and barriers to work and also to take advantage of facilitators (such as personal factors and workplace factors). Inter-sectoral collaboration and social resources are important (Fig. 1). Health professionals (e.g., occupational health professionals and vocational rehabilitation staff) who are responsible for a system to support individuals with chronic diseases returning and continuing to work are often not experts for specific diseases. However, previous studies were limited to particular diseases, such as cancer, and they focused on those conditions. Our results can apply to various physical diseases. We identified barriers and facilitators in terms of 10 sub-themes, which can help health professionals facilitate support.

We categorized the barriers and facilitators for individuals with physical diseases to return to and continue working into three themes (personal factors, workplace factors, and inter-sectoral collaboration and social resources) and 10 sub-themes (work ability, psychological impacts, health literacy, social status, family background, workplace structure, workplace system, workplace support, inter-sectoral collaboration, and social resources). We believe our results offer a better understanding of the various barriers and facilitators for employees with physical diseases such that they can be addressed at medical institutions, workplaces, and other support sites. Our results provide a foundation for better communicating with individuals and facilitate their returning and continuing to work. This study contributes to achieving the SDG target of 8.5, which is full and productive employment and decent work for all women and men, including for young people and persons with disabilities, and equal pay for work of equal value.

## Data Availability

The datasets used and/or analyzed during the current study are available from the corresponding author on reasonable request.

## Abbreviations

SDGs: Sustainable development goals
CRT-D: Cardiac resynchronization therapy-defibrillator
ICD: Implantable cardioverter defibrillator
PTCA: Percutaneous transluminal coronary angioplasty
CT: Chemotherapy
RT: Radiotherapy
BMT: Bone marrow transplantation
(M): Manager
OECD: Organisation for economic co-operation and development
